# Synergistic Regulation of Active Phases, Carbon Matrix, and Electron Transport in CNT-Incorporated Electrospun Co-Mo/P Polyacrylonitrile-Derived Carbon Nanofibers for Alkaline Hydrogen Evolution

**DOI:** 10.3390/polym18202598

**Published:** 2026-10-21

**Authors:** Xiuhong Li, Xin Jin, Libo Wang, Zhiyong Yang, Jinjiao Zhang, Xinyu Hu, Daode Zhang, Yong Liu, Yan Chen

**Affiliations:** 1 Hubei Key Laboratory of Modern Manufacturing Quality Engineering, School of Mechanical Engineering, Hubei University of Technology, Wuhan 430068, China; 20200005@hbut.edu.cn (X.L.); 102510194@hbut.edu.cn (X.J.); 102410058@hbut.edu.cn (L.W.); yzy017@126.com (Z.Y.); 19940054@hbut.edu.cn (J.Z.); 19991012@hbut.edu.cn (X.H.); hgzdd@126.com (D.Z.); 2 School of Materials Science and Engineering, Beijing University of Chemical Technology, Chaoyang District, Beijing 100029, China; yongliu@mail.buct.edu.cn; 3 School of Mechanical and Electrical Engineering, Wuhan Donghu University, Wuhan 430212, China

**Keywords:** electrospun nanofibers, Co-Mo carbides, P incorporation, thermal treatment, carbon nanotubes, alkaline hydrogen evolution reaction

## Abstract

Sluggish alkaline hydrogen evolution reaction (HER) kinetics restrict large-scale applications of non-noble-metal carbon-based electrocatalysts. Herein, CNT-incorporated Co-Mo/P polyacrylonitrile-derived carbon nanofibers were prepared by electrospinning followed by thermal treatment (pre-oxidation and carbonization), where the Co/Mo feed molar ratio, thermal treatment conditions, and CNT content were sequentially optimized. A continuous and uniform polyacrylonitrile-derived carbon nanofiber matrix was obtained, where pre-oxidation helped preserve the fibrous framework during carbonization. Active phases associated with metallic Co, Mo_2_C, and Co_3_Mo_3_C were identified and P-M (M = Co or Mo) bonding was found. Furthermore, conductive networks were formed via doping CNTs in the electrospun Co-Mo/P-based electrocatalyst, enhancing their electron transport. The optimized sample exhibited HER activity with overpotentials of 106 and 312 mV at 10 and 100 mA cm^−2^ as well as a Tafel slope of 99.1 mV dec^−1^ in 1 mol L^−1^ KOH electrolyte. These results demonstrate that the coordinated regulation of active phases, carbon matrix, and electron transport improved the alkaline HER performance of the electrospun Co-Mo/P-based electrocatalyst. This synergistic strategy may offer a potential pathway for developing an efficient, robust, and cheap electrocatalyst for alkaline HER.

## 1. Introduction

Hydrogen is an important energy carrier with a high gravimetric energy density, which can bridge the generation, storage, and end use of renewable electricity, making it a key component of the transition toward a low-carbon energy system [1,2,3]. Among the various hydrogen-production routes, water electrolysis has attracted considerable attention owing to its compatibility with renewable electricity, high hydrogen purity, and low operational carbon emissions [4,5]. Alkaline water electrolysis offers several advantages, including technological maturity, compatibility with non-noble-metal electrode materials, and suitability for large-scale production [6,7]. However, HER at the cathode is generally limited by the sluggish and complex reaction kinetics [8,9,10]. The alkaline HER involves water activation, the adsorption and conversion of reaction intermediates, and interfacial water reorganization [11,12,13,14]. Although Pt-based materials remain the benchmark electrocatalysts for the HER because of their favorable hydrogen adsorption properties, the high cost and rare natural abundance hinder their large-scale applications in alkaline water electrolysis [15,16,17]. Thus, developing efficient, stable, and low-cost non-noble-metal electrocatalysts that can promote water dissociation, appropriately regulate H adsorption, and facilitate electron transport is essential for improving the efficiency of alkaline HER [18,19,20].

As non-noble-metal HER electrocatalysts, transition-metal carbides have attracted considerable attention due to their tunable metal–carbon bonding, good electrical conductivity, and high chemical stability [21,22,23,24]. Usually, transition-metal carbides are applied as nanoparticles in HER electrocatalysts [25,26]. However, these nanoscale carbide particles are prone to migration, grain growth, and agglomeration during their synthesis under high-temperature conditions [22]. As a result, suitable support architectures are essential for their confinement and stabilization, which can be realized via electrospinning and subsequent pre-oxidation and carbonization for a composite of polymers and transition-metal carbide precursors. Electrospinning can enable polymers, metal precursors, and other functional components to be uniformly integrated into continuous nanofibers [27,28]. Then, subsequent pre-oxidation and carbonization of the composite allow active phases to be formed and anchored in situ within carbon nanofiber frameworks [25,26,29]. The resulting carbon nanofibers can suppress active-phase agglomeration, provide continuous electron-transport pathways, and improve the accessibility of catalytic sites [30]. The Co-Mo-based catalytic system is one of the most popular transition-metal carbide electrocatalysts. The interfacial interactions between Co- and Mo-based carbides can induce local charge redistribution, thereby affecting water dissociation, H adsorption, and interfacial electron transfer [31,32]. Various Co-Mo-based catalytic systems containing Co/MoC, Co/Mo_2_C, Mo_2_C-CoNi, Co_6_Mo_6_C_2_-Mo_2_C, and P-Co_3_Mo_3_C/Co have been constructed in electrospun CNFs, which have shown promising HER or overall water-splitting performance [33,34,35,36]. Nevertheless, incorporating Co-Mo-based active phases into electrospun carbon nanofibers cannot guarantee high electrocatalytic activity. The Co/Mo composition influences active-phase formation and the interfacial electronic structure [32,34,37], while P incorporation can further regulate the carbide composition and local coordination environment [36,38,39,40,41]. Meanwhile, the pre-oxidation and carbonization of polymers determine fiber structural integrity, defect retention, and carbon structural ordering [29,42]. In addition to the above-mentioned characteristics, the performance of electrospun Co-Mo-based electrocatalysts is also related to the overall conductivity of the fibrous network, which depends on inter-fiber connectivity [43]. This can be optimized by introducing an appropriate amount of CNTs with controlled content and dispersion, which can improve the structural ordering and electron-transport capability of electrospun CNFs. Therefore, the HER capability of electrospun Co-Mo-based CNF electrocatalysts relies on the rational matching of the active phases, carbon matrix, and electron transport.

The HER performance of electrospun Co-Mo-based electrocatalysts has been increased through strategies such as metal composition and coordination environment regulation, heterointerface construction, nonmetal doping and defect engineering, thermal treatment conditions, and integration with conductive carbon frameworks [42,43,44,45]. For example, some studies combined active-phase regulation with carbon-support design to increase the catalytic ability of electrospun Co-Mo-based electrocatalysts, where Co-Mo sulfide heterojunctions [46], Co-Mo metal clusters [47], Co-Co_6_Mo_6_C_2_ heterostructures [48], and Co/Mo dual-atom sites [49] have been constructed within electrospun carbon nanofibers to simultaneously regulate active-site dispersion, interfacial coupling, and electron-transport behavior. In addition, approaches involving N-doped Co_6_Mo_6_C and Co/Mo-N coordination [50,51], P vacancies [52], Co-Mo phosphide heterointerfaces [53,54,55], amorphous Co-Mo-B phases [56], and Co-regulated Mo_2_C [57,58] have been employed to optimize the local electronic structures, reaction-intermediate adsorption, and interfacial charge transfer to enhance catalytic performance. But most previous studies related to electrospun Co-Mo-based electrocatalysts for the HER have focused on only one or two aspects among the active phases, carbon matrix, and electron transport to adjust their catalytic abilities. Moreover, some regulation methods are often implemented using different material compositions and preparation conditions, making it difficult to compare the structural changes and performance changes induced by each regulation step on a unified basis. In particular, systematic studies on sequentially regulating the precursor composition, thermal-treatment conditions, and CNT content of electrospun Co-Mo-based CNF electrocatalysts based on the same preparation platform remain limited.

Inspired by the above considerations, a high-performance Co-Mo/P-CNF doped with CNTs as an HER electrocatalyst was investigated in this study through electrospinning followed by thermal treatment (pre-oxidation and carbonization). To establish the synergistic regulation of the active phases, carbon matrix, and electron transport for enhancing the alkaline HER performance of the electrospun Co-Mo/P carbon nanofiber, the effects of precursor composition and P-source introduction, thermal treatment conditions, and CNT content on the electrocatalytic properties were sequentially evaluated. The experimental results demonstrated that the optimized Co-Mo/P-CNF-41-750-CNT2 electrocatalyst exhibited the optimum alkaline HER capability. This research provides a basis for coordinating active-phase formation, carbon-matrix evolution, and electron-transport behavior in alkaline HER electrocatalysis, showcasing the promising potential for designing non-noble-metal HER electrocatalysts.

## 2. Experimental Section

### 2.1. Materials

Polyacrylonitrile (PAN, *M*_w_ = 150,000 g/mol), cobalt acetate tetrahydrate (99.5%), phosphomolybdic acid hydrate of analytical reagent (AR) grade, molybdenum acetylacetonate (Mo(acac)_3_, ≥98%), and N,N-dimethylformamide (DMF, 99.5%) were supplied by Shanghai Macklin Biochemical Co., Ltd. (Shanghai, China). CNTs (diameter: 3–15 nm, length: 15–30 μm, purity: >98%) were acquired from Shenzhen Suiheng Technology Co., Ltd.(Shenzhen, China). Potassium hydroxide (KOH, AR grade) was supplied by Sinopharm Chemical Reagent Co., Ltd. (Shanghai, China). Super P Li conductive carbon black was obtained from Guangdong Canrd New Energy Technology Co., Ltd. (Guangdong, China). Nafion solution (D520, 5 wt%) was supplied by Suzhou Zhengtairong Technology Co., Ltd. (Suzhou, China). Nickel foam (thickness: 1 mm, pore density: 110 ppi) was purchased from Suzhou Keshenghe Metal Materials Co., Ltd. (Suzhou, China). High-purity argon (Ar, ≥99.999%) was supplied by Wuhan Zhongxinhua Technology Co., Ltd. (Wuhan, China). All the chemicals were used without further purification.

### 2.2. Preparation of Electrocatalysts

#### 2.2.1. Electrospinning of Co-Mo/P and Co-Mo Precursor Nanofibers

Co-Mo/P precursor nanofibers with different Co/Mo feed molar ratios were prepared by electrospinning. First, 1.113 g of PAN was dissolved in 10 mL of DMF under magnetic stirring at room temperature until complete dissolution. Subsequently, 0.071 g of phosphomolybdic acid hydrate was added as Mo and P sources, together with 0.249, 0.374, 0.498, or 0.623 g of cobalt acetate tetrahydrate as the Co source. The resulting solutions were continuously stirred for 12 h to obtain homogeneous electrospinning solutions with Co/Mo feed molar ratios of approximately 2:1, 3:1, 4:1, and 5:1, respectively, as calculated from the precursor amounts. The electrospinning solutions were loaded into a syringe equipped with a 23 G needle and then electrospun at an applied voltage of 20 kV, a solution feed rate of 0.7 mL h^−1^, a tip-to-collector distance of 20 cm, and a collector rotating speed of 800 rpm. The ambient temperature and relative humidity were maintained at approximately 25 °C and 40%, respectively. After electrospinning, the precursor nanofibers were collected for subsequent thermal treatment.

For the P-free reference series, Mo(acac)_3_ was employed as the Mo source in place of phosphomolybdic acid hydrate. The amount of Mo(acac)_3_ was fixed at 0.197 g, while 0.249, 0.374, 0.498, or 0.623 g of cobalt acetate tetrahydrate was added to get Co/Mo feed molar ratios of approximately 2:1, 3:1, 4:1, and 5:1, respectively. The amount of the other materials (PAN, DMF, etc.), the solution preparation procedure, and electrospinning conditions were kept the same as those used for the P-containing series.

#### 2.2.2. Thermal Treatment of Electrospun Precursor Nanofibers

The thermal treatment consists of two steps: pre-oxidation and carbonization. Initially, the electrospun precursor nanofibers were pre-oxidized in a muffle furnace at 250 °C in air for 90 min at a heating rate of 5 °C min^−1^ and were placed between two quartz plates to minimize warping and deformation during pre-oxidation. After natural cooling to room temperature, the pre-oxidized electrospun precursor nanofibers were transferred to a tube furnace and carbonized at 750 °C under Ar for 2 h at the same heating rate, followed by natural cooling to room temperature. The resulting P-containing samples with Co/Mo feed molar ratios of approximately 2:1, 3:1, 4:1, and 5:1 were denoted as Co-Mo/P-CNF-21-750, Co-Mo/P-CNF-31-750, Co-Mo/P-CNF-41-750, and Co-Mo/P-CNF-51-750, respectively. The corresponding P-free samples were named Co-Mo-CNF-21-750, Co-Mo-CNF-31-750, Co-Mo-CNF-41-750, and Co-Mo-CNF-51-750, respectively.

To investigate the effects of carbonization temperature on the electrocatalytic performance of the samples, Co-Mo/P precursor nanofibers with a Co/Mo feed molar ratio of approximately 4:1 were pre-oxidized at 250 °C in air for 90 min at a heating rate of 5 °C min^−1^ and subsequently carbonized at 650, 700, 750, 800, or 850 °C under Ar for 2 h at the same heating rate, yielding Co-Mo/P-CNF-41-650, Co-Mo/P-CNF-41-700, Co-Mo/P-CNF-41-750, Co-Mo/P-CNF-41-800, and Co-Mo/P-CNF-41-850, respectively.

Similarly, to evaluate the influence of pre-oxidation on the electrocatalytic performance of the samples, Co-Mo/P precursor nanofibers with a Co/Mo feed molar ratio of approximately 4:1 were directly carbonized at 650, 700, 750, 800, or 850 °C under Ar for 2 h at a heating rate of 5 °C min^−1^ without pre-oxidation. The obtained samples were referred to as Co-Mo/P-CNF-41-u650, Co-Mo/P-CNF-41-u700, Co-Mo/P-CNF-41-u750, Co-Mo/P-CNF-41-u800, and Co-Mo/P-CNF-41-u850, respectively, where u denotes the absence of pre-oxidation.

#### 2.2.3. Preparation of CNT-Incorporated Co-Mo/P-CNF Composites

For the CNT-containing series, 10, 20, and 30 mg of CNTs were separately added to Co-Mo/P electrospinning solutions with a Co/Mo feed molar ratio of approximately 4:1. The resulting CNT contents were approximately 0.9, 1.8, and 2.7 wt% relative to the mass of PAN, respectively. The CNT-containing electrospinning solutions were ultrasonically dispersed using an ultrasonic power of 80 W for 1 h and then magnetically stirred at 60 °C and 400 rpm for 8 h. The final solutions were electrospun under the same conditions as those described in Section 2.2.1. And the obtained CNT-containing electrospun precursor nanofibers were subsequently pre-oxidized at 250 °C in air for 90 min and carbonized at 750 °C under Ar for 2 h with the same heating rate of 5 °C min^−1^, resulting in Co-Mo/P-CNF-41-750-CNT1, Co-Mo/P-CNF-41-750-CNT2, and Co-Mo/P-CNF-41-750-CNT3, respectively.

### 2.3. Materials Characterization

The morphology and fibrous structure of the samples were examined using scanning electron microscopy (SEM, SU8010, Hitachi, Tokyo, Japan). The elemental composition and spatial distribution of the electrospun precursor fibers were analyzed using energy-dispersive X-ray spectroscopy (EDS). The crystal structures and phase compositions of the samples were characterized via X-ray diffraction (XRD, Empyrean Alpha 1, PANalytical, Almelo, The Netherlands) with Cu Kα radiation at an operating voltage of 40 kV over a 2θ range of 10–90°. The elemental compositions and chemical states of the samples were indicated by X-ray photoelectron spectroscopy (XPS, PHI 5000 VersaProbe I, PHI, Chigasaki, Japan). The defect characteristics and structural ordering of the carbon matrix were evaluated using a confocal Raman microscope (XploRA Plus, HORIBA, Palaiseau, France) with an excitation wavelength of 532 nm, where the intensity ratio of the D and G bands (*I*_D_/*I*_G_) was used to assess the structural ordering degree of the carbon matrix.

### 2.4. Electrochemical Measurements

All electrochemical measurements were conducted on a CHI 660E electrochemical workstation with a conventional three-electrode configuration, where a graphite rod, an Hg/HgO electrode, and a nickel foam loaded with the electrocatalyst were used as the counter, reference, and working electrodes, respectively. The electrolyte was a 1 mol L^−1^ KOH aqueous solution. The measured potential was converted to the reversible hydrogen electrode (RHE) scale, and the current density was normalized to the geometrically exposed area of the working electrode.

All the working electrodes were prepared by the same procedure. First, 14 mg of the thoroughly ground carbonized nanofiber membranes and 6 mg of carbon black were dispersed in a solvent containing 14 mL of deionized water, 6 mL of isopropanol, and 40 μL of 5 wt% Nafion solution. The mixture was ultrasonicated for 30 min to obtain a homogeneous catalytic ink. Then, an appropriate amount of the ink was uniformly drop-cast onto pretreated nickel foam with an effective area of 1 cm × 1 cm, followed by vacuum drying at 80 °C for 12 h.

Linear sweep voltammetry (LSV) was used to evaluate the HER performance of the electrocatalyst over a potential range from 0.1 to −0.5 V vs. RHE at a scan rate of 1 mV s^−1^. All polarization curves were corrected for 90% of the uncompensated ohmic potential drop (*iR*). The catalytic activity was analyzed by the overpotentials required to reach current densities of 10 and 100 mA cm^−2^, which are denoted as *η*_10_ and *η*_100_, respectively. The Tafel slope was obtained by fitting the polarization curves using Equation (1):

(1) η =a + blog|j|,

where η is the overpotential, *a* is the intercept, *b* is the Tafel slope, and |j| is the absolute value of the current density.

The *C*_dl_ was determined from cyclic voltammetry (CV) measurements conducted within a non-Faradaic potential region at scan rates of 20, 40, 60, 80, and 100 mV s^−1^. The anodic current density (*j*_a_) and cathodic current density (*j*_c_) were recorded at a selected potential, and the half-difference in current density was calculated according to Equation (2):

(2) $$\Delta j\;=\;(j_{a}\;-\;j_{c})/2.$$

The *C*_dl_ value was the slope of the linear relationship between Δ*j* and the scan rate, which was employed to compare the relative changes in the electrochemically accessible surface area of different samples under identical measured conditions.

Electrochemical impedance spectroscopy (EIS) was performed at −0.15 V vs. RHE over a frequency range from 100 kHz to 0.1 Hz, with an alternating-current perturbation amplitude of 5 mV. The Nyquist plots were fitted using an equivalent circuit consisting of the solution resistance (*R*_s_), interfacial charge-transfer resistance (*R*_ct_), and a constant-phase element (CPE), where *R*_ct_ and CPE are connected in parallel, with *R*_s_ connected in series. The fitted *R*_ct_ values were used to compare the interfacial charge-transfer characteristics of the catalysts.

## 3. Results and Discussion

### 3.1. Structural Characterization

#### 3.1.1. Morphological and Elemental Characterization of Electrospun Co-Mo/P Precursor Nanofibers

**Figure 1:**
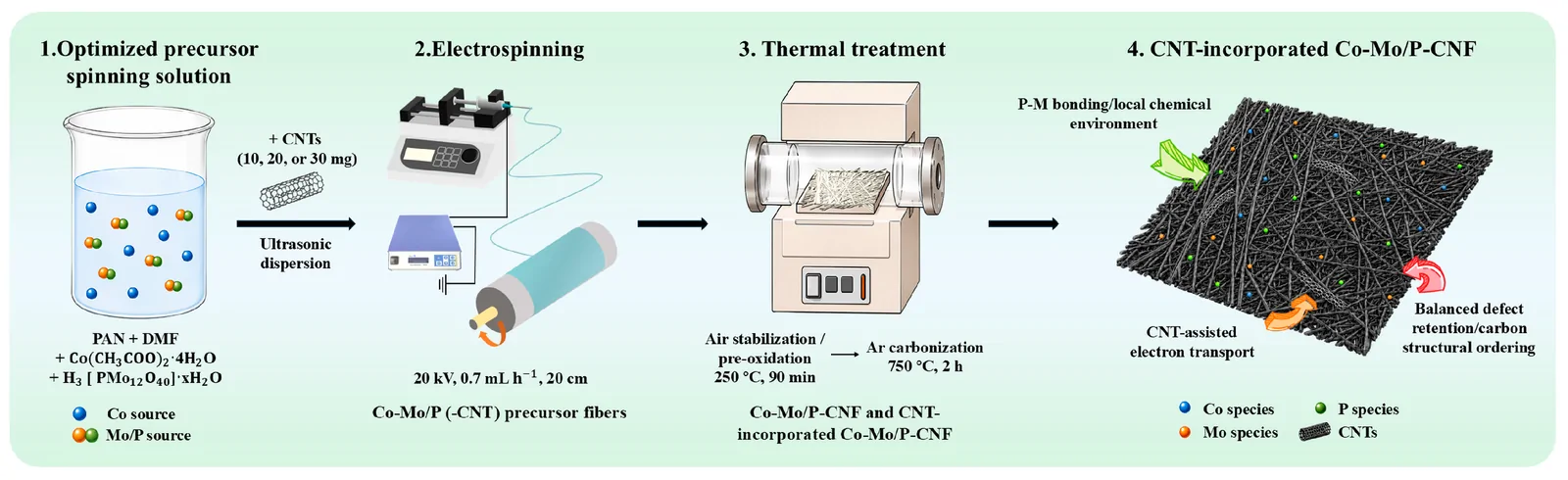
Schematic illustration of the preparation procedure of electrospun Co-Mo/P-CNF and Co-Mo/P-CNF-CNT electrocatalysts.

The preparation procedures of the electrospun Co-Mo/P-CNF and Co-Mo/P-CNF-CNT electrocatalysts are schematically illustrated in Figure 1, which sequentially focuses on the Co/Mo feed molar ratio, thermal-treatment conditions, and CNT content. Thus, the active phases, carbon-matrix structures, and electron transport of the composite fibrous membrane can be gradually regulated, affecting the electrocatalytic performance of the obtained electrocatalysts.

**Figure 2:**
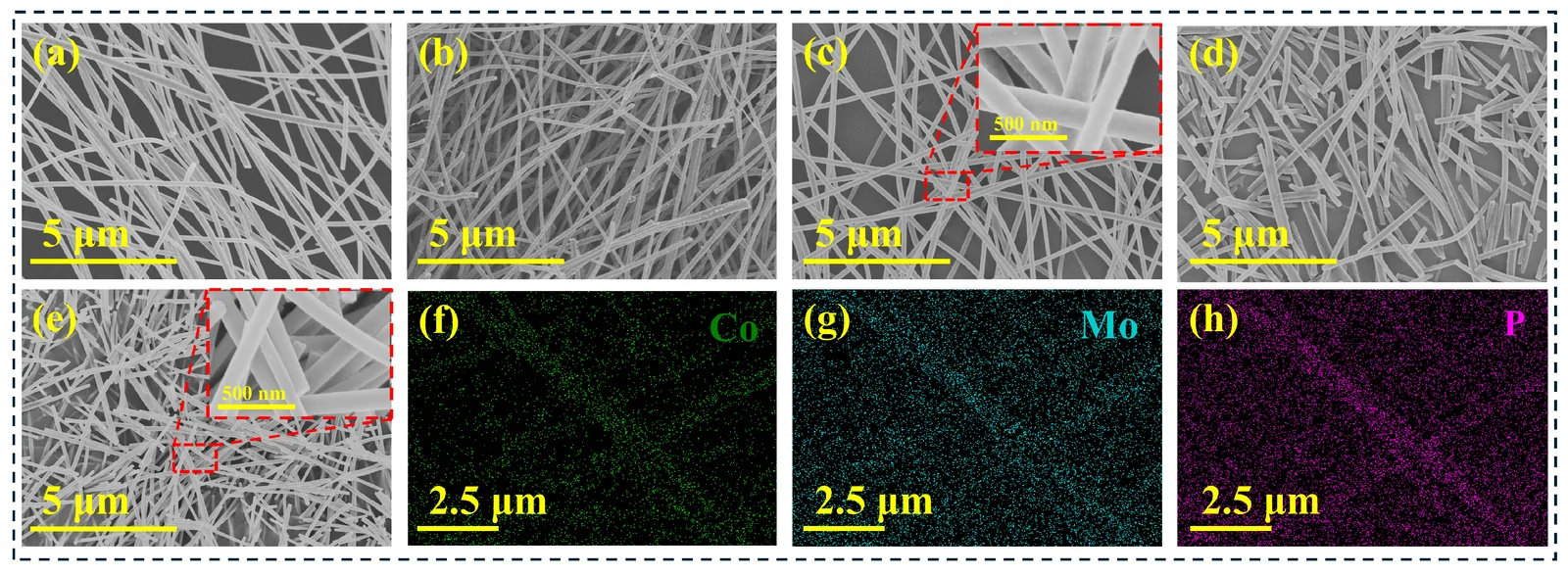
Morphological and elemental characterization of Co-Mo/P and Co-Mo precursor nanofibers. (**a**–**d**) SEM images of the P-containing precursor nanofibers with Co/Mo feed molar ratios of 2:1, 3:1, 4:1, and 5:1, respectively. (**e**) SEM image of the P-free precursor nanofibers with a Co/Mo feed molar ratio of 4:1. The insets in (**c**) and (**e**) show the corresponding high-magnification SEM images. (**f**–**h**) EDS mappings of Co, Mo, and P in the P-containing precursor nanofibers with a Co/Mo feed molar ratio of 4:1.

Figure 2a–d shows the SEM images of the resulting electrospun precursor fibrous membranes at different precursor compositions, where the Co/Mo feed molar ratios are 2:1, 3:1, 4:1, and 5:1, respectively. Observation reveals that the fibers in all four samples exhibit uniform and continuous morphologies with random orientations, although their continuity, diameter uniformity, and degree of local breakage differ. At a Co/Mo feed molar ratio of 4:1, the precursor nanofibers were generally continuous, relatively uniform in diameter, and smooth in surface morphology, indicating favorable fiber-forming characteristics. Based on the high-magnification inset in Figure 2c, the fiber diameters of Co-Mo/P-CNF-41 were estimated to be approximately 200 nm. In contrast, when the Co/Mo feed molar ratio deviated from 4:1, more obvious diameter variations and local fiber breakage were observed, proving that the precursor composition had a pronounced influence on the fiber formation, continuity, and uniformity during electrospinning.

As shown in Figure 2e and its inset, the P-free precursor nanofibers with a Co/Mo feed molar ratio of 4:1 also retained a well-defined morphology. A further comparison of the high-magnification SEM images of the P-containing and P-free samples showed that both types of precursor nanofibers exhibited good continuity and uniformity, demonstrating that the introduction of the P source had no effect on the fiber formation (insets in Figure 2c,e). To examine the spatial distribution of the precursor components, EDS elemental mapping was performed on the Co-Mo/P-CNF-41 precursor nanofibers before carbonization. As illustrated in Figure 2f–h, Co, Mo, and P were relatively uniformly distributed throughout the fiber regions, without obvious large-scale elemental segregation. This result indicates that the precursor components were relatively homogeneously dispersed within the PAN fiber matrix during electrospinning.

#### 3.1.2. Structural and Surface Chemical Characterization of Carbonized Co-Mo/P-CNF

**Figure 3:**
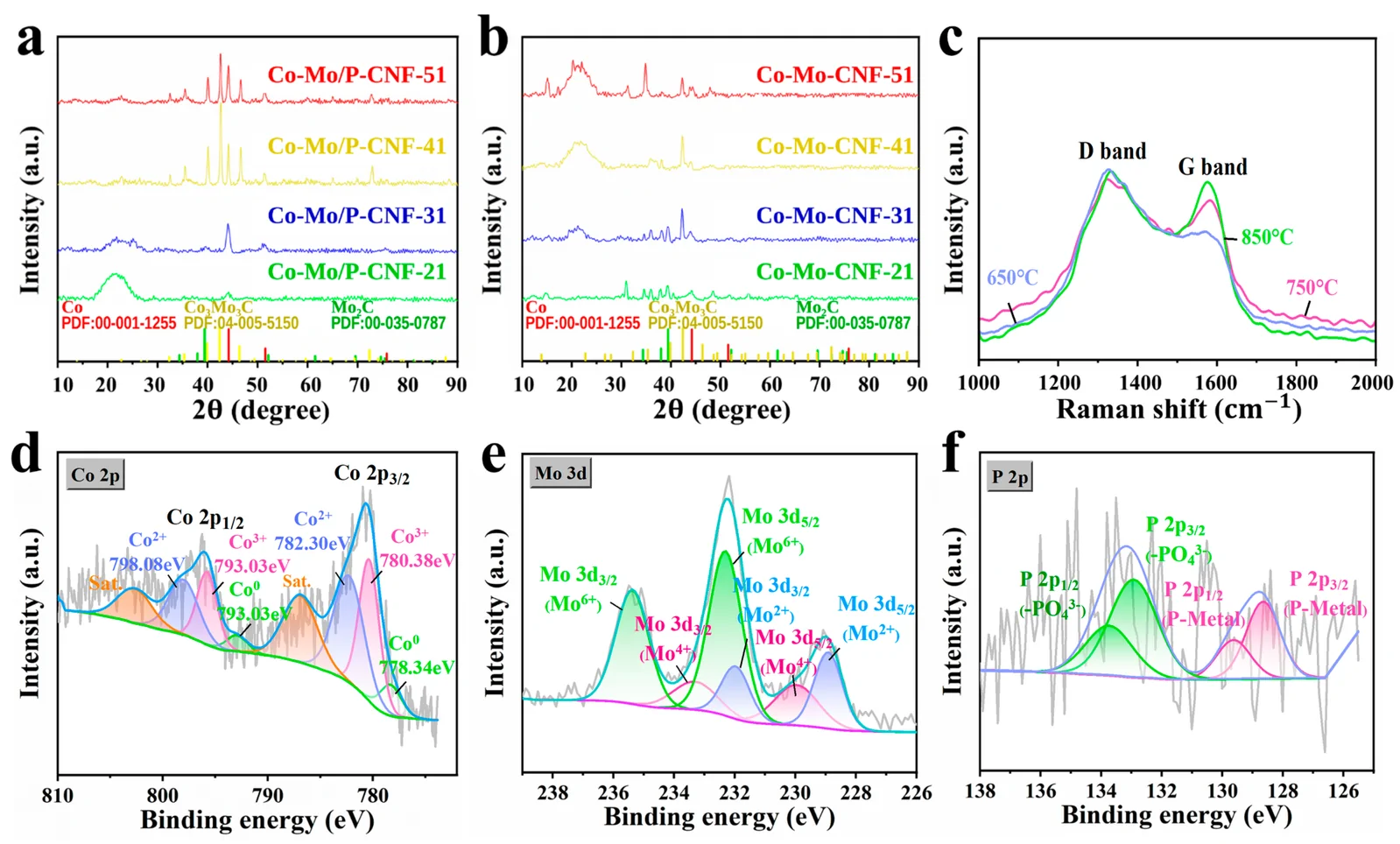
Phase composition, carbon structural ordering, and surface chemical states of the Co-Mo/P-CNF and Co-Mo-CNF samples. (**a**,**b**) XRD patterns of the P-containing and P-free samples with different Co/Mo feed molar ratios, respectively. (**c**) Raman spectra of Co-Mo/P-CNF-41 samples carbonized at 650, 750, and 850 °C. (**d**–**f**) High-resolution Co 2p, Mo 3d, and P 2p XPS spectra of Co-Mo/P-CNF-41-750, respectively. The standard PDF card numbers are metallic Co (PDF No. 00-001-1255), Mo_2_C (PDF No. 00-035-0787), and Co_3_Mo_3_C (PDF No. 04-005-5150).

Then, the obtained precursor nanofibers with different Co/Mo feed molar ratios were subjected to thermal treatments at various carbonization temperatures, with and without pre-oxidation, described in detail in Section 2.2. To study the effects of the precursor composition, especially the introduction of P, on the formation and crystallization behaviors of Co-Mo carbides during thermal treatment, the crystalline phases of the carbonized samples were first analyzed by XRD. As shown in Figure 3a, the samples with different Co/Mo feed molar ratios under pre-oxidation and carbonization at 750 °C exhibited characteristic diffraction peaks associated with metallic Co, Mo_2_C, and Co_3_Mo_3_C. The diffraction features related to Co_3_Mo_3_C were more pronounced in the samples with Co/Mo feed molar ratios of 4:1 and 5:1. Specifically, the diffraction peaks at approximately 44.2°, 51.5°, and 75.9° could be assigned to metallic Co, whereas those at approximately 34.4°, 38.0°, 39.4°, 52.1°, 61.5°, 69.6°, and 74.6° corresponded to Mo_2_C. The peaks at approximately 32.3°, 35.3°, 39.9°, 42.4°, and 46.4° were consistent with Co_3_Mo_3_C [31,36]. Variations in the relative intensities of these diffraction peaks among the samples indicated that the precursor composition affected the formation and crystallization behaviors of the Co-Mo carbides during thermal treatment.

To primarily investigate the influence of P on the phase composition of the resulting samples, the XRD patterns of the P-free Co-Mo-CNF series were further compared. As shown in Figure 3b, diffraction features associated with metallic Co, Mo_2_C, and Co_3_Mo_3_C were also found in the P-free reference samples. However, the peaks related to Mo_2_C and Co_3_Mo_3_C were generally weaker than those in the P-containing samples. The differences between the P-containing phosphomolybdic acid [H_3_P(Mo_3_O_10_)_4_·xH_2_O] system and the P-free molybdenum acetylacetonate [Mo(acac)_3_] reference system suggested that the introduction of the P source together with the accompanying changes in the precursor may affect the formation and crystallization of Co-Mo carbides. It should be noted that XRD peak intensities can also be affected by their crystallite sizes, degree of crystallinity, and preferred orientations. Therefore, these results were examined primarily to identify the phase composition and its relative variation in the above-mentioned samples.

Meanwhile, the structural evolution of the electrospun Co-Mo/P carbon matrix at different carbonization temperatures was further investigated by Raman spectroscopy. To compare the evolution of the carbon matrix with carbonization temperature, three representative samples carbonized at 650, 750, and 850 °C were selected for Raman analysis. The following discussion focuses on the electrospun Co-Mo/P carbonized at 650, 750, and 850 °C. As shown in Figure 3c, all samples exhibited D and G bands at approximately 1324–1332 and 1570–1582 cm^−1^, respectively, which are related to defective and disordered carbon structures, whereas the G band originates from the in-plane stretching vibration of sp^2^-hybridized carbon atoms [42,43]. For the samples carbonized at 650, 750, and 850 °C, the D-band peaks were located at approximately 1327, 1324, and 1332 cm^−1^, respectively, while the corresponding G-band peaks appeared at approximately 1570, 1582, and 1575 cm^−1^. These slight shifts in peak positions reflect changes in the carbon structure. A comparison of the Raman spectra showed that Co-Mo/P-CNF-41-650 exhibited a relatively high defect density but a comparatively low degree of carbon ordering, whereas Co-Mo/P-CNF-41-850 possessed a more ordered carbon structure accompanied by a lower defect density. By comparison, Co-Mo/P-CNF-41-750 achieved a more reasonable balance between defect retention and carbon structural ordering. Additionally, the *I*_D_/*I*_G_ values of Co-Mo/P-CNF-41-650, Co-Mo/P-CNF-41-750, and Co-Mo/P-CNF-41-850 were approximately 1.6, 1.3, and 0.9, respectively. It is suggested that the *I*_D_/*I*_G_ of the samples after thermal treatment decreased with increasing carbonization temperature, indicating a gradual enhancement in the structural ordering of the carbon matrix [42]. From these findings, it is demonstrated that the best carbonization temperature of the electrospun Co-Mo/P nanofibers was 750 °C and proper carbonization temperature is helpful for maintaining superior defect and carbon structural ordering.

The surface chemical states of Co-Mo/P-CNF-41-750 were further investigated by high-resolution XPS. As shown in Figure 3d, the Co 2p component at approximately 778.3 eV was assigned to metallic Co, whereas the higher-binding-energy components were assigned to Co^2+^ and Co^3+^ species, accompanied by characteristic shake-up satellite features [31,36], suggesting the presence of partially oxidized Co species on the sample surface. In the Mo 3d spectrum, the doublet at approximately 228.5 and 231.7 eV was assigned to low-valence Mo species associated with Mo–C bonding. The Mo^4+^ and Mo^6+^ components at higher binding energies were mainly related to partial oxidation of the sample surface (Figure 3e) [25,38]. In the P 2p spectrum, the low-binding-energy doublet at approximately 128.6 and 129.4 eV was attributed to metal-P bonding species, denoted as P-M (M = Co or Mo), whereas the higher-binding-energy components were mainly associated with P-O bonds or oxidized P species on the surface (Figure 3f) [36,38,39]. The XRD results suggest that the P-containing precursor influenced the Co–Mo carbide formation, while XPS indicates the presence of P–M (M = Co or Mo) bonding. These findings are consistent with previous reports on P-mediated regulation of the local coordination and electronic structures of transition metals [36,39]. The Co 2p and Mo 3d XPS spectra demonstrate metallic Co, Mo–C bonding, and oxidized Co/Mo species near the surface, whereas XRD identifies the crystalline phases of Mo_2_C and Co_3_Mo_3_C in the bulk, indicating that the surface chemical environment relevant to HER may differ from the bulk phase composition.

The high-resolution C 1s spectrum of Co-Mo/P-CNF-41-750 is shown in Figure S6. The peaks at 284.80, 286.12, and 287.87 eV correspond to C–C/C=C, C–O, and C=O bonds, respectively. The dominant C–C/C=C component (82.33% of the fitted C 1s peak area) indicates that carbon–carbon bonding is the main surface carbon chemical state detected by XPS.

#### 3.1.3. Morphological Evolution and Structural Integrity of Co-Mo/P-CNF

**Figure 4:**
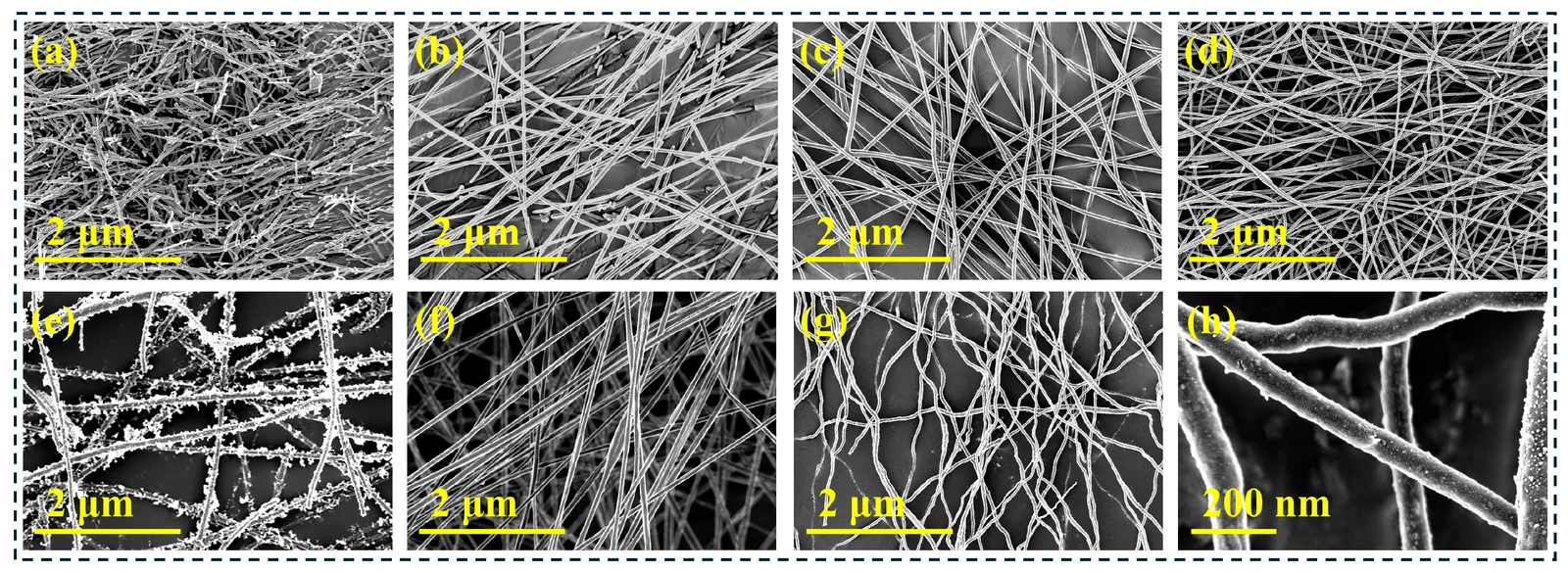
Effects of thermal treatment and CNT incorporation on the morphology of Co-Mo/P-CNF. (**a**–**e**) SEM images of Co-Mo/P-CNF-41 samples pre-oxidized at 250 °C and carbonized at 650, 700, 750, 800, and 850 °C, respectively. (**f**) SEM image of Co-Mo/P-CNF-41-u750 prepared without pre-oxidation. (**g**,**h**) Low- and high-magnification SEM images of Co-Mo/P-CNF-41-750-CNT2.

According to the above discussion, the optimal precursor composition is the Co/Mo feed molar ratio of 4:1. Thus, the structural evolution of the electrospun composite fibers under the thermal treatment, including pre-oxidation and carbonization, was studied based on this composition. It is noted that the thermal treatment conditions markedly affected the fibrous structure of the electrospun Co-Mo/P nanofibers [29,42]. As shown in Figure 4a–e, all samples subjected to pre-oxidation at 250 °C retained fibrous morphologies after carbonization at different temperatures, although clear differences in fiber continuity and surface characteristics were observed. Co-Mo/P-CNF-41-650 showed extensive fiber breakage, indicating that a sufficiently stable fibrous framework had not yet developed at the relatively low carbonization temperature. As the carbonization temperature reached 700 and 750 °C, the fiber continuity improved markedly and the fiber surfaces became relatively smooth. In particular, Co-Mo/P-CNF-41-750 displayed a comparatively uniform and intact fibrous network. While the carbonization temperature was further increased to 800 and 850 °C, pronounced particle precipitation and local aggregation appeared on the fiber surfaces, which may be associated with the migration of metal species and grain growth at elevated temperatures [22]. These results revealed that an appropriate carbonization temperature is beneficial for maintaining fiber integrity and suppressing the excessive aggregation of active phases.

To examine the effects of pre-oxidation on the structure of electrospun Co-Mo/P nanofibers, the Co-Mo/P-CNF-41 was directly carbonized at 750 °C without pre-oxidation, which is noted as Co-Mo/P-CNF-41-u750. As shown in Figure 4f, Co-Mo/P-CNF-41-u750 presented obvious fiber breakage, adhesion, and local fusion. Its structural integrity was substantially lower than that of the pre-oxidized Co-Mo/P-CNF-41-750. As a result, thermally stable ladder structures would gradually form in the electrospun composite, which can mitigate melting, shrinkage, and deformation of the fibers during subsequent high-temperature carbonization [29,42]. Therefore, pre-oxidation plays an important role in preserving and enhancing the thermal stability of the fibrous framework.

It is known that CNT incorporation has been widely used to improve the electrical conductivity and electron-transport capability of carbon-based materials, which may be beneficial for enhancing their electrocatalytic performance [43]. Hence, to further optimize the electrocatalytic ability of the obtained Co-Mo/P-based electrocatalyst, CNTs with contents of 0.9, 1.8, and 2.7 wt% relative to the mass of PAN were separately added to the electrospun Co-Mo/P precursor solution with the optimal Co-Mo feed molar ratio. Then, the resulting samples were subjected to the best thermal treatment conditions, which are denoted as Co-Mo/P-CNF-41-750-CNT1, Co-Mo/P-CNF-41-750-CNT2, and Co-Mo/P-CNF-41-750-CNT3, respectively. CNT-free Co-Mo/P-CNF-41-750 was used as the reference (Figure 4c). According to the electrochemical test results of the Co-Mo/P-CNF-41-750 doped with CNTs discussed in Section 3.2, Co-Mo/P-CNF-41-750-CNT2 exhibited superior electrocatalytic activity.

The low- and high-magnification images of Co-Mo/P-CNF-41-750-CNT2 are illustrated in Figure 4g,h. It is observed that the sample retained an interlaced fibrous network with good overall continuity. No extensive fiber fusion, network collapse, or severe fracture was identified, indicating that the fibrous structure was preserved after pre-oxidation and carbonization. Compared with the CNT-free Co-Mo/P-CNF-41-750, the fibers in the sample doped with CNTs appeared less straight and showed more pronounced local bending, waviness, and entanglement. These changes suggested that CNT incorporation influenced the local arrangement and morphological evolution of the electrospun Co-Mo/P composite fibers during thermal treatment.

The high-magnification image of Co-Mo/P-CNF-41-750-CNT2 (Figure 4h) further shows that the individual nanofibers remained continuous, without obvious cracking or extensive breakage. This demonstrates that added CNTs would not induce global instability of the electrospun Co-Mo/P fibrous framework during the thermal treatment. Plus, local surface irregularities and slight bending were also observed. This is attributed to the different mechanical rigidity and thermal shrinkage behavior of CNTs and PAN-derived carbon, which may produce local variations in stress and shrinkage during pre-oxidation and carbonization [43,59].

Both of the SEM images confirmed that Co-Mo/P-CNF-41-750-CNT2 kept the overall integrity of the fibrous network while exhibiting more pronounced local bending and entanglement. Such morphological changes may alter the contact between adjacent fibers and provide a structural basis for electron transport. However, conventional SEM mainly reveals the surface morphology of the composite fibers and cannot directly determine the distribution of CNTs within the carbon nanofibers. Nor can these images alone verify the internal connectivity of CNTs within the composite fibers. Therefore, the specific roles of CNTs in the electrocatalytic process must be assessed with the following electrochemical tests.

### 3.2. Electrochemical Performance

**Figure 5:**
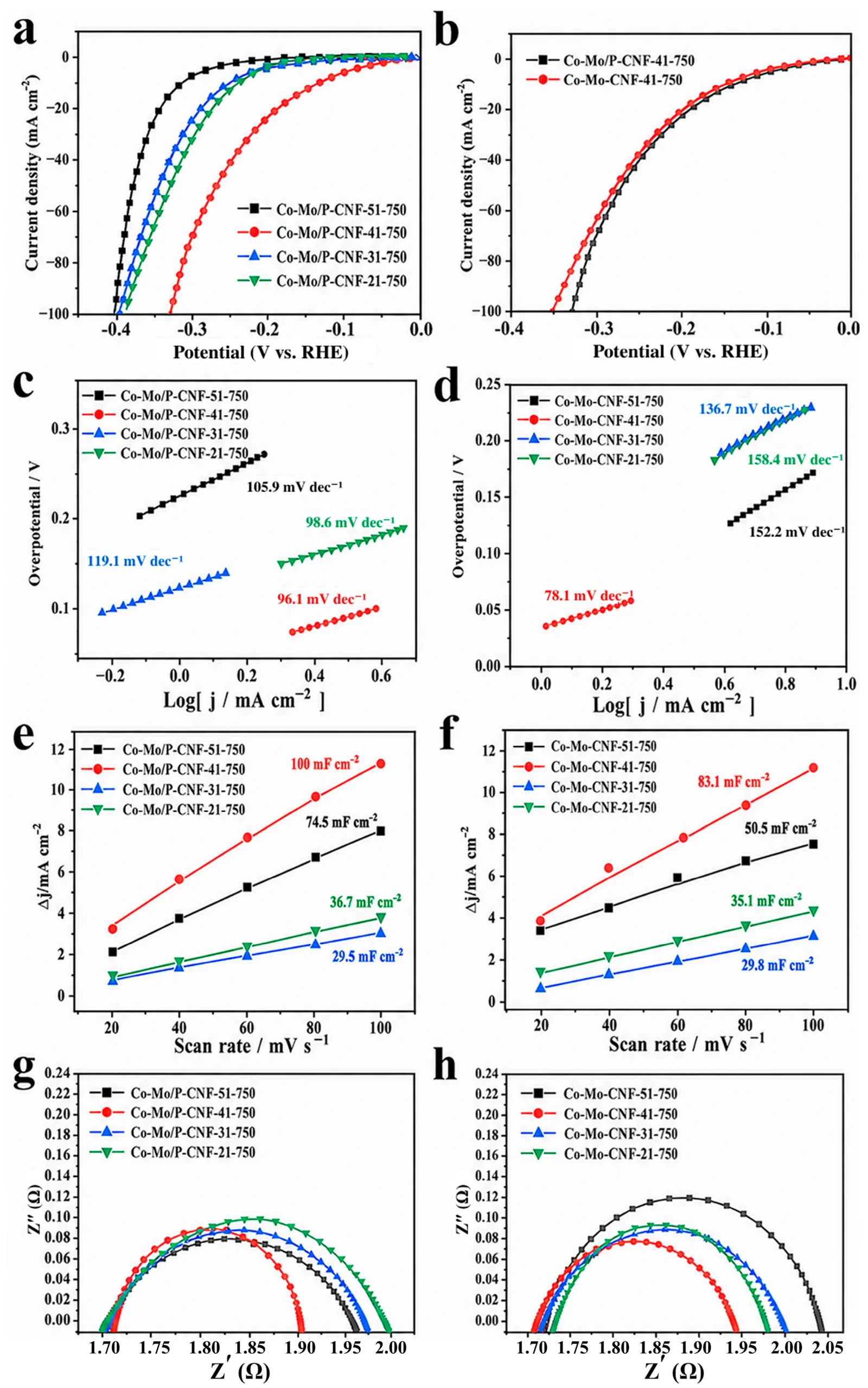
Effects of the Co/Mo feed molar ratio and introduction of the P source on alkaline HER performance. (**a**) *iR*-corrected LSV curves of the P-containing samples with different Co/Mo feed molar ratios. (**b**) *iR*-corrected LSV curves of Co-Mo/P-CNF-41-750 and Co-Mo-CNF-41-750. (**c**,**d**) Tafel plots, (**e**,**f**) *C*_dl_ fitting plots, and (**g**,**h**) Nyquist plots of the P-containing and P-free sample series, respectively. All samples were pre-oxidized at 250 °C and carbonized at 750 °C.

The alkaline HER performances of all samples were evaluated in 1 mol L^−1^ KOH aqueous solution using *iR*-corrected LSV curves, Tafel slopes, double-layer capacitances, and electrochemical impedance measurements to analyze their electrocatalytic activity and interfacial kinetics. In terms of the results obtained in the structural characterization section, it is noted that pre-oxidation at 250 °C and carbonization at 750 °C are the optimal thermal-treatment conditions. Accordingly, the following discussions depend on this. The effect of the Co/Mo feed molar ratio on the alkaline HER performance of the Co-Mo/P-CNF samples was first investigated. As shown in Figure 5a, the samples prepared with different Co/Mo feed molar ratios exhibited distinctly different polarization behaviors. Among them, Co-Mo/P-CNF-41-750 required the overpotentials of 137 and 328 mV to reach current densities of 10 and 100 mA cm^−2^, respectively. These values were lower than those of Co-Mo/P-CNF-21-750 (245 and 390 mV), Co-Mo/P-CNF-31-750 (242 and 395 mV), and Co-Mo/P-CNF-51-750 (313 and 401 mV). These results demonstrate that the Co/Mo feed molar ratio markedly affected the alkaline HER activity of the samples, where a precursor composition with a Co/Mo feed molar ratio of 4:1 was more favorable for achieving improved electrocatalytic performance.

Similarly, the impacts of P-source introduction on the electrocatalytic activity of the Co-Mo/P-CNF-41-750 were examined. Figure 5b shows the LSV of Co-Mo/P-CNF-41-750 and the P-free Co-Mo-CNF-41-750. It can be observed that the overpotentials required for the P-free sample were 148 and 351 mV at 10 and 100 mA cm^−2^, respectively, whereas the corresponding values for the P-containing sample were reduced to 137 and 328 mV. The lower overpotentials of the latter suggest that the P-containing system displayed more favorable alkaline HER activity than the P-free reference system. This is also confirmed by the Tafel analysis of the P-containing and P-free sample series with various Co/Mo feed molar ratios shown in Figure 5c and Figure 5d, respectively. Both of the two series revealed that the Co/Mo feed molar ratio of 4:1 showed the lowest overpotential to get the set current density. Within the P-containing series, Co-Mo/P-CNF-41-750 possessed a Tafel slope of 96.1 mV dec^−1^, which was lower than those of Co-Mo/P-CNF-31-750 and Co-Mo/P-CNF-51-750 and comparable to that of Co-Mo/P-CNF-21-750. However, the P-free Co-Mo-CNF-41-750 sample exhibited a Tafel slope of 78.1 mV dec^−1^, which was lower than that of the P-containing sample. According to the Tafel equation, the overpotential depends on both the Tafel slope and intercept. Reanalysis of Co-Mo/P-CNF-41-750 yielded a slope of 96.09 mV dec^−1^, an intercept of 24.88 mV, and R^2^ = 0.9990, which are consistent with the reported Tafel slope of 96.1 mV dec^−1^. The effects of electrochemically accessible surface area and interfacial charge transfer on HER performance are further discussed below based on the *C*_dl_ and EIS results.

The *C*_dl_ outcomes further reflect differences in the electrochemically accessible surface areas of the samples with several Co/Mo feed molar ratios. The corresponding CV curves used to determine *C*_dl_ for the P-free and P-containing series are provided in Figure S1 and Figure S2, respectively. As depicted in Figure 5e, Co-Mo/P-CNF-41-750 exhibited a *C*_dl_ value of 100 mF cm^−2^, higher than those of the other samples in the P-containing series. In the P-free series, Co-Mo-CNF-41-750 showed a *C*_dl_ value of 83.1 mF cm^−2^, which is also the highest among them (Figure 5f). In addition, the *C*_dl_ value of the P-containing sample with a molar ratio of 4:1 was approximately 20% higher than that of the P-free reference sample. Although *C*_dl_ is not equivalent to the absolute electrochemically active surface area, it can be used to indirectly compare the electrochemically accessible surface area among the electrodes measured under identical conditions [60]. Consequently, these results indicated that the P-containing sample possessed a relatively larger electrochemically accessible surface area, showing better electrocatalytic activity.

Likewise, EIS measurements also revealed differences in interfacial charge transfer between the P-containing system and the P-free reference system, where Co-Mo/P-CNF-41-750 and Co-Mo-CNF-41-750 displayed the lowest *R*_ct_. As shown in Figure 5g,h, Co-Mo/P-CNF-41-750 exhibited an *R*_ct_ value of 0.166 Ω, which is lower than the 0.249 Ω measured for the P-free Co-Mo-CNF-41-750 reference sample, suggesting a decreased *R*_ct_ for the P-containing sample. Together with the phase-composition and surface-chemical-state results discussed in structure characterization in Section 3.1, the improved HER activity of the P-containing system may be associated with modification of the local chemical environment of the Co-Mo carbides, enlargement of the electrochemically accessible surface area, and improved interfacial charge transfer. This corresponds to previous studies that P can regulate the electronic and coordination environments of transition metals and facilitate interfacial charge transfer [38,39]. Nevertheless, the decrease in overpotential caused by P-source introduction was not accompanied by a corresponding reduction in the Tafel slope. Therefore, the performance enhancement cannot be simply attributed to accelerated Tafel kinetics. All in all, the above-mentioned analysis demonstrated that the Co/Mo molar ratio of 4:1 is the preferred precursor composition.

**Figure 6:**
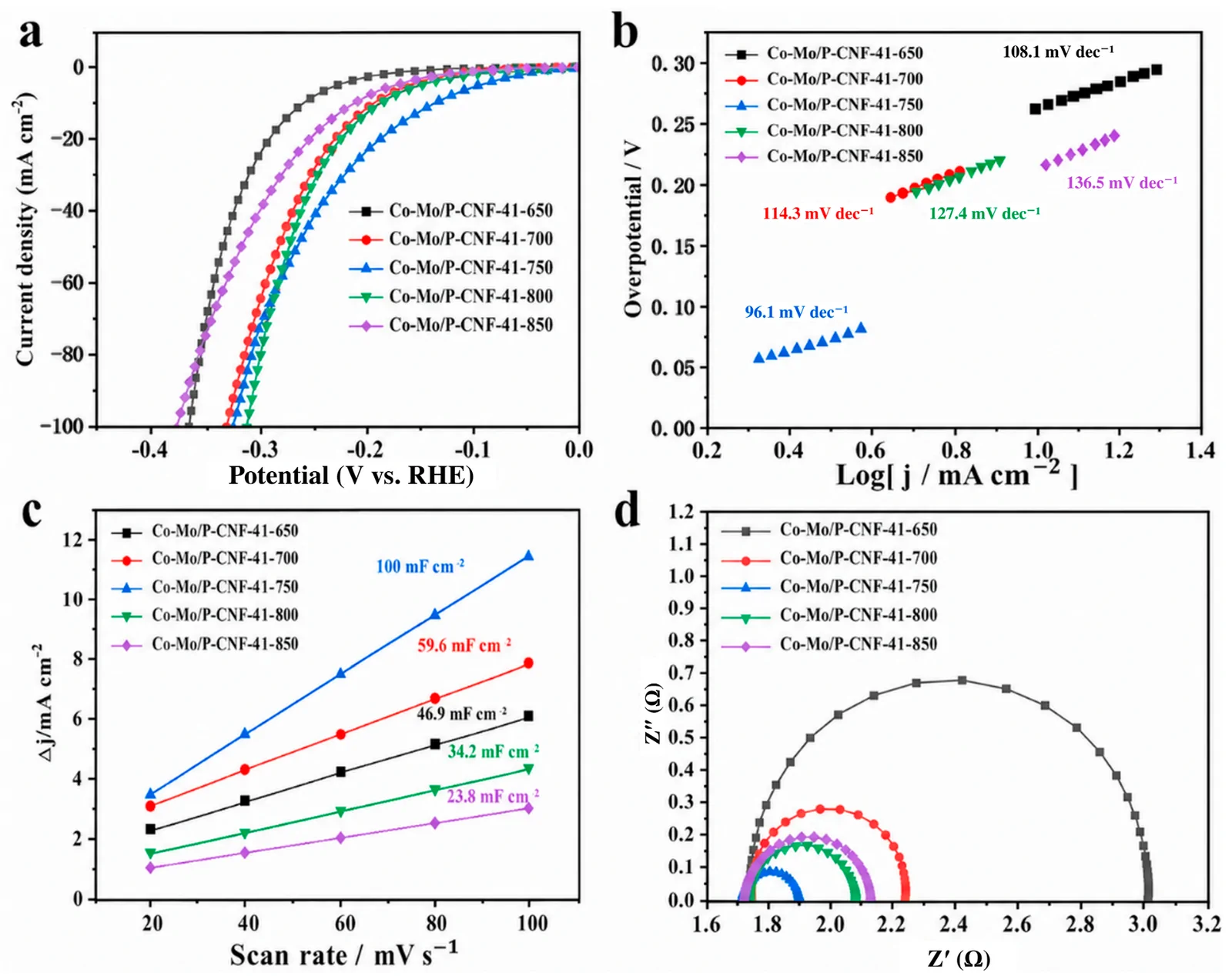
Effect of carbonization temperature on the alkaline HER performance of Co-Mo/P-CNF-41. (**a**) iR-corrected LSV curves, (**b**) Tafel plots, (**c**) C_dl_ fitting plots, and (**d**) Nyquist plots of the samples carbonized at different temperatures.

Accordingly, the effects of thermal treatment on the HER performance of the Co-Mo/P-CNF samples were studied. As shown in Figure 6a, the LSV trends of the samples after pre-oxidation at 250 °C exhibited a non-monotonic dependence of activity on the carbonization temperature. As the carbonization temperature increased, the overpotentials required for the samples at 10 and 100 mA cm^−2^ first decreased and then increased. Co-Mo/P-CNF-41-750 had the lowest overpotential of 137 mV at 10 mA cm^−2^, while its overpotential at 100 mA cm^−2^ was 328 mV, slightly higher than that of Co-Mo/P-CNF-41-800. Thus, the sample carbonized at 750 °C showed the highest electrocatalytic activity at the lower current density, whereas the sample carbonized at 800 °C showed a slight advantage at the higher current density. The results indicate that the influence of carbonization temperature on HER performance cannot be evaluated from the overpotential at a single current density alone and requires further comparison of HER kinetics, electrochemically accessible surface area, and interfacial charge-transfer behavior.

Furthermore, Tafel analysis also confirmed the effects of carbonization temperature on the HER kinetics of the samples. As shown in Figure 6b, the Tafel slopes of the samples carbonized at 650, 700, 750, 800, and 850 °C were 108.1, 114.3, 96.1, 127.4, and 136.5 mV dec^−1^, respectively. Co-Mo/P-CNF-41-750 possessed the lowest Tafel slope, demonstrating more favorable HER kinetics within the carbonization-temperature series. The corresponding *C*_dl_ results showed the same change, in that Co-Mo/P-CNF-41-750 reached the largest *C*_dl_ value of 100 mF cm^−2^, which is substantially higher than the values of 46.9, 59.6, 34.2, and 23.8 mF cm^−2^ obtained for the samples carbonized at 650, 700, 800, and 850 °C, respectively (Figure 6c). The corresponding CV curves used to determine *C*_dl_ are provided in Figure S3. This revealed that the sample carbonized at a temperature of 750 °C possessed the largest electrochemically accessible surface area within this series.

Meanwhile, Co-Mo/P-CNF-41-750 had an *R*_ct_ value of 0.166 Ω, exhibiting the smallest impedance semicircle in the thermal-treatment comparison series, as illustrated in Figure 6d. This value was smaller than those of Co-Mo/P-CNF-41-650, Co-Mo/P-CNF-41-700, Co-Mo/P-CNF-41-800, and Co-Mo/P-CNF-41-850, which were 1.281, 0.500, 0.346, and 0.414 Ω, respectively. It is also validated that a carbonization temperature of 750 °C was more favorable for interfacial charge transfer. All of the corresponding electrochemical parameters for the Co-Mo/P-CNF-41 samples carbonized at different temperatures are summarized in Table 1.

The EIS fitting χ^2^ values for Co-Mo/P-CNF-41-650, -700, -750, -800, and -850 were 8.4056 × 10^−5^, 7.4787 × 10^−5^, 4.3603 × 10^−5^, 6.9130 × 10^−5^, and 8.1832 × 10^−5^, respectively, with corresponding residuals of 2.75%, 2.55%, 1.59%, 2.48%, and 2.67%. The 650 °C sample showed a Nyquist semicircle diameter of approximately 1.3 Ω, consistent with its fitted R_ct_ of 1.281 Ω. Its larger semicircle may be related to lower carbon structural ordering at 650 °C, as suggested by the Raman results. The 750 °C sample showed the lowest χ^2^ and residual among the five samples.

Combined with the SEM and Raman results shown in the previous Section 3.1, the influences of carbonization temperatures on the alkaline HER performance can be attributed to the combined effects of fiber structural integrity, defect retention, and carbon structural ordering. The sample carbonized at 650 °C showed a relatively high defect density but a comparatively low degree of carbon ordering, which may limit electron transport in the electrocatalytic process. In general, defects in carbon materials can provide potential electrocatalytic or anchoring sites, whereas a higher degree of graphitization favors electron conduction [42,43]. As a result, the Co-Mo/P-CNF-41-650 had poor electrocatalytic activity. When the carbonization temperature was increased to 750 °C, a more reasonable balance among fiber structural integrity, defect retention, and carbon structural ordering was achieved, resulting in a larger electrochemically accessible surface area and a lower *R*_ct_. Although the carbon structures became more ordered, the particulate precipitation and aggregation on the fiber surfaces grew more pronounced, together with the markedly decreased *C*_dl_. This is consistent with previous reports that elevated temperatures promote metal-particle migration and sintering, leading to a decline in overall electrochemical performance [22]. Considering the overpotentials, Tafel slopes, *C*_dl_, and EIS results, 750 °C was proved to be the most appropriate carbonization temperature for the subsequent CNT-incorporation study.

In addition, pre-oxidation also had an obvious impact on alkaline HER activity. Co-Mo/P-CNF-41-u750 directly carbonized at 750 °C without pre-oxidation required an overpotential of 234 mV at 10 mA cm^−2^, which was profoundly higher than that of the pre-oxidized Co-Mo/P-CNF-41-750 sample. Though its *C*_dl_ can reach 95.9 mF cm^−2^, its *R*_ct_ also increased to 0.255 Ω. The complete electrochemical comparison of the samples directly carbonized without pre-oxidation at 650–850 °C is provided in Figure S4, while low- and corresponding high-magnification SEM images of the samples obtained at 650, 700, 800, and 850 °C are provided in Figure S5. Together with the fiber breakage, adhesion, and local fusion observed in Figure 4f, these results demonstrated that the beneficial effect of pre-oxidation on HER performance did not arise solely from an enhancement in the electrochemically accessible surface area, but was also closely related to improved fiber structural stability and interfacial charge-transfer conditions [29,42].

On the basis of the optimized precursor composition of the Co/Mo feed molar ratio and the thermal-treatment conditions, the effects of CNT content on the alkaline HER performance of the Co-Mo/P-CNF composite electrocatalysts were continuously explored. As listed in Table 2, the CNT-free Co-Mo/P-CNF-41-750 sample required overpotentials of 137 and 328 mV at 10 and 100 mA cm^−2^, respectively, while the overpotentials required for Co-Mo/P-CNF-41-750-CNT1, Co-Mo/P-CNF-41-750-CNT2, and Co-Mo/P-CNF-41-750-CNT3 were 119, 106, 106, 329, 312, and 309 mV at 10 mA cm^−2^ and 100 mA cm^−2^, respectively. Increasing the CNT content from 0.9 to 1.8 wt% reduced the polarization behavior at both lower and higher current densities. However, when the CNT content was increased to 2.7 wt%, the overpotential at 10 mA cm^−2^ did not decrease further, and only a slight reduction in the overpotential at 100 mA cm^−2^ was observed. It is demonstrated that an appropriate CNT content was beneficial for enhancing the alkaline HER activity of the Co-Mo/P-CNF composites, whereas further increasing the CNT content provided only limited additional improvement.

**Figure 7:**
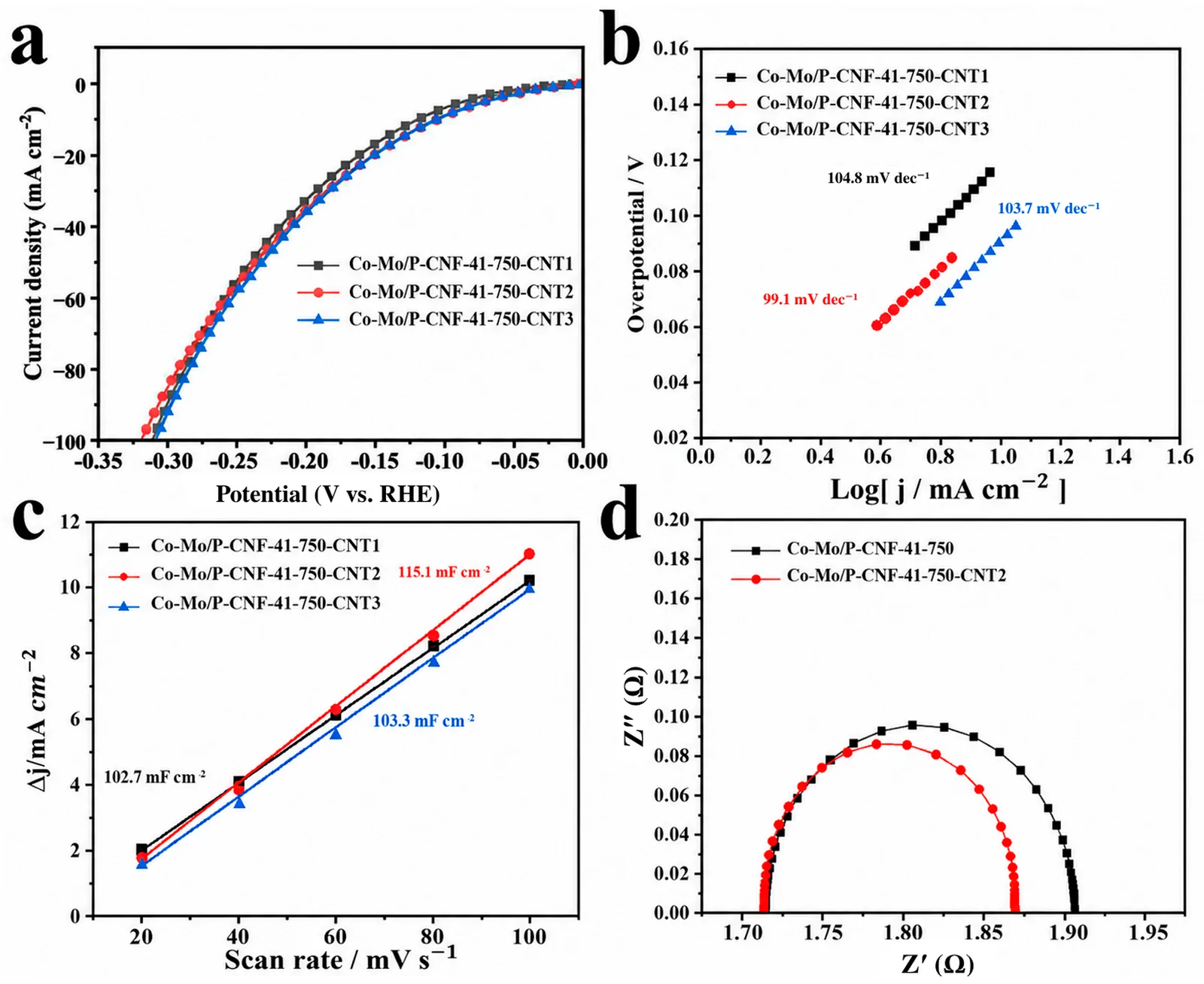
Effect of CNT content on the alkaline HER performance of Co-Mo/P-CNF-41-750 composites. (**a**) iR-corrected LSV curves, (**b**) Tafel plots, and (**c**) C_dl_ fitting plots of the CNT-containing samples. (**d**) Nyquist plots of Co-Mo/P-CNF-41-750 and Co-Mo/P-CNF-41-750-CNT2.

As shown in Figure 7b, the Tafel slopes of Co-Mo/P-CNF-41-750-CNT1, Co-Mo/P-CNF-41-750-CNT2, and Co-Mo/P-CNF-41-750-CNT3 were 104.8, 99.1, and 103.7 mV dec^−1^, respectively. Among them, Co-Mo/P-CNF-41-750-CNT2 exhibited the lowest Tafel slope. For comparison, the CNT-free sample showed a Tafel slope of 96.1 mV dec^−1^ (Table 2). Therefore, although Co-Mo/P-CNF-41-750-CNT2 had a lower overpotential, its Tafel slope was not lower than that of the CNT-free sample, which indicates that the lower overpotentials may also be related to improved electrochemical accessibility and charge transfer.

The *C*_dl_ results further manifested the influence of CNT content on the electrochemically accessible surface area. As illustrated in Figure 7c, the *C*_dl_ values of Co-Mo/P-CNF-41-750-CNT1, Co-Mo/P-CNF-41-750-CNT2, and Co-Mo/P-CNF-41-750-CNT3 were 102.7, 115.1, and 103.3 mF cm^−2^, respectively. Co-Mo/P-CNF-41-750-CNT2 possessed the largest *C*_dl_ value, approximately 15% higher than the value of 100 mF cm^−2^ obtained for the CNT-free sample, indicating that an appropriate CNT content improved the electrochemically accessible surface area. When the CNT content was further increased to 2.7 wt%, *C*_dl_ decreased to 103.3 mF cm^−2^, showing that additional CNT incorporation did not continuously enlarge the effective electrochemically accessible surface area. This behavior may be associated with changes in the composite fibrous structure and CNT dispersion at higher CNT contents [43].

Moreover, the Nyquist plots showed that Co-Mo/P-CNF-41-750-CNT2 exhibited a smaller impedance semicircle than the CNT-free Co-Mo/P-CNF-41-750 sample (Figure 7d), demonstrating that CNT incorporation enhanced the interfacial charge transfer. Coupled with the findings in Section 3.1, an appropriate amount of CNTs may help establish more continuous electron-transport pathways among the carbon nanofibers, thereby facilitating interfacial charge transfer and increasing the electrochemically accessible surface area [21,43].

Considering the overpotentials, Tafel slopes, *C*_dl_, and EIS results, Co-Mo/P-CNF-41-750-CNT2 displayed the most balanced overall electrochemical performance. Although Co-Mo/P-CNF-41-750-CNT3 showed a slightly lower overpotential than Co-Mo/P-CNF-41-750-CNT2 at 100 mA cm^−2^, it possessed a lower *C*_dl_ and a higher Tafel slope. Together, the lower Tafel slope and higher C_dl_ support selecting Co-Mo/P-CNF-41-750-CNT2 as the preferred sample among the investigated CNT contents.

To further evaluate the HER performance of the selected Co-Mo/P-CNF-41-750-CNT2, Table 3 compares it with previously reported Co–Mo-based and P-containing electrocatalysts, as well as literature-reported Pt/C and RuAu_3_ benchmarks. The optimized electrocatalyst exhibits an η_10_ of 106 mV and a Tafel slope of 99.1 mV dec^−1^ in 1 mol L^−1^ KOH. Notably, its overpotential is comparable to that of CoMoS@CNF (105.2 mV) [46], indicating competitive HER activity. This comparison highlights the potential of the electrospun Co–Mo/P-CNF system as a non-noble-metal electrocatalyst for alkaline HER.

Overall, the P-containing sample with a Co/Mo feed molar ratio of 4:1 exhibited more favorable interfacial properties and lower HER overpotentials. Pre-oxidation followed by carbonization at 750 °C further established a balance among fiber structural integrity, defect retention, and carbon structural ordering. On this basis, incorporation of 1.8 wt% CNTs increased the electrochemically accessible surface area and improved charge transfer, reducing *η*_10_ from 137 to 106 mV. These observations demonstrated that the sequential optimization of the precursor composition, Co/Mo feed molar ratio, thermal-treatment conditions, and CNT content enhanced the alkaline HER performance of the electrospun Co-Mo/P carbon nanofibers through regulation of active phases, carbon matrix, and electron transport, respectively.

## 4. Conclusions

In summary, Co-Mo/P fibrous electrocatalysts with synergistic regulation of active phases, carbon matrix, and electron transport were constructed via electrospinning followed by thermal treatment. By sequentially exploring the effects of precursor composition, thermal-treatment conditions, and CNT content on the electrocatalytic properties of the electrospun Co-Mo/P carbon nanofibers, their active-phase components, carbon-matrix structure, and electron-transport network were progressively optimized. Structural characterization and electrochemical measurements demonstrated that the electrospun precursor nanofibers with a Co-Mo feed molar ratio of 4:1 showed the best favorable microstructures and electrocatalytic characteristics. The P-containing system exhibited better electrocatalytic features than the P-free system. Additionally, pre-oxidation helped preserve the structural integrity of the fibers during high-temperature carbonization, while carbonization at 750 °C achieved a reasonable balance among fiber structural integrity, defect retention, and carbon structural ordering. Furthermore, the incorporation of 1.8 wt% CNTs can improve the electrochemically accessible surface area and interfacial charge transfer of the resulting sample. These results revealed that the synergistic regulation of active phases, carbon matrix, and electron transport via controlling the precursor composition, thermal-treatment conditions, and CNT content of the electrospun Co-Mo/P carbon nanofibers can enhance their alkaline HER performance, holding promise for creating non-noble-metal electrocatalysts for alkaline HER.

## Figures and Tables

**Table 1:** Alkaline HER electrochemical parameters of Co-Mo/P-CNF-41 samples carbonized at different temperatures.

Sample	*η*_10_ (mV)	*η*_100_ (mV)	Tafel Slope (mV dec^−1^)	*C*_dl_ (mF cm^−2^)	*R*_ct_ (Ω)
Co-Mo/P-CNF-41-650	263	369	108.1	46.9	1.281
Co-Mo/P-CNF-41-700	196	335	114.3	59.6	0.500
Co-Mo/P-CNF-41-750	137	328	96.1	100	0.166
Co-Mo/P-CNF-41-800	191	316	127.4	34.2	0.346
Co-Mo/P-CNF-41-850	213	381	136.5	23.8	0.414

**Table 2:** Alkaline HER electrochemical parameters of Co-Mo/P-CNF-41-750 samples with different CNT contents.

Sample	CNT Content Relative to PAN (wt%)	*η*_10_ (mV)	*η*_100_ (mV)	Tafel Slope (mV dec^−1^)	*C*_dl_ (mF cm^−2^)
Co-Mo/P-CNF-41-750	0	137	328	96.1	100
Co-Mo/P-CNF-41-750-CNT1	0.9	119	329	104.8	102.7
Co-Mo/P-CNF-41-750-CNT2	1.8	106	312	99.1	115.1
Co-Mo/P-CNF-41-750-CNT3	2.7	106	309	103.7	103.3

**Table 3:** Comparison of HER performance between the electrocatalyst prepared and previously reported.

Catalyst	*η*_10_ (mV)	*η*_100_ (mV)	Tafel Slope (mV dec^−1^)	Electrolyte	Ref.
Co-Mo/P-CNF-41-750-CNT2	106	312	99.1	1 mol L^−1^ KOH	This work
CoMoS@CNF	105.2	—	152.8	1 mol L^−1^ KOH	[46]
CoMo/CN@CNFs/MEL	148	—	98.85	1 mol L^−1^ KOH	[47]
Co-Co_6_Mo_6_C_2_/CNFs	91	—	—	Alkaline electrolyte	[48]
Co_2_Mo_2_-NPCNFs	—	259.8	—	1 mol L^−1^ KOH	[49]
CoMoP/Cr (OH)_3_/NF	88	—	—	1 mol L^−1^ KOH	[54]
Co-Mo-B/NF	—	199	141	Alkaline seawater	[56]
Co/Mo_2_C@N-CKNs	98	—	—	1 mol L^−1^ KOH	[57]
Pt/C (20 wt%, literature)	43	—	54.2	1 mol L^−1^ KOH	[61]
RuAu_3_ aerogel	24.1	—	36.7	1 mol L^−1^ KOH	[62]

Note: “—” denotes unavailable values.

## Data Availability

The original contributions presented in this study are included in the article. Further inquiries can be directed to the corresponding author.

## Supplementary Materials

The following supporting information can be downloaded at: https://www.mdpi.com/article/10.3390/polym18202598/s1, Figure S1: Cyclic voltammetry (CV) curves of P-free Co-Mo-CNF samples with different Co/Mo feed molar ratios; Figure S2: Cyclic voltammetry (CV) curves of P-containing Co-Mo/P-CNF samples with different Co/Mo feed molar ratios; Figure S3: Cyclic voltammetry (CV) curves of pre-oxidized Co-Mo/P-CNF-41 samples carbonized at different temperatures; Figure S4: Full electrochemical comparison of Co-Mo/P-CNF-41 samples directly carbonized without pre-oxidation at 650–850 °C; Figure S5: Low-magnification SEM images and their corresponding high-magnification images of Co-Mo/P-CNF-41 samples directly carbonized without pre-oxidation at different temperatures; Figure S6: High-resolution XPS C 1s spectrum of Co-Mo/P-CNF-41-750, including fitted carbon-bonding components.
