# Supplementary material for: Synergistic Regulation of Active Phases, Carbon Matrix, and Electron Transport in CNT-Incorporated Electrospun Co-Mo/P Polyacrylonitrile-Derived Carbon Nanofibers for Alkaline Hydrogen Evolution
Source: Polymers (Basel). 2026 Oct 21;18(20):2598. doi: 10.3390/polym18202598 (PMC13708907; doi:10.3390/polym18202598)
Supplement: Supplementary file 1 [file polymers-18-02598-s001.zip › polymers-4576981-supplementary.pdf]

Supplementary Materials

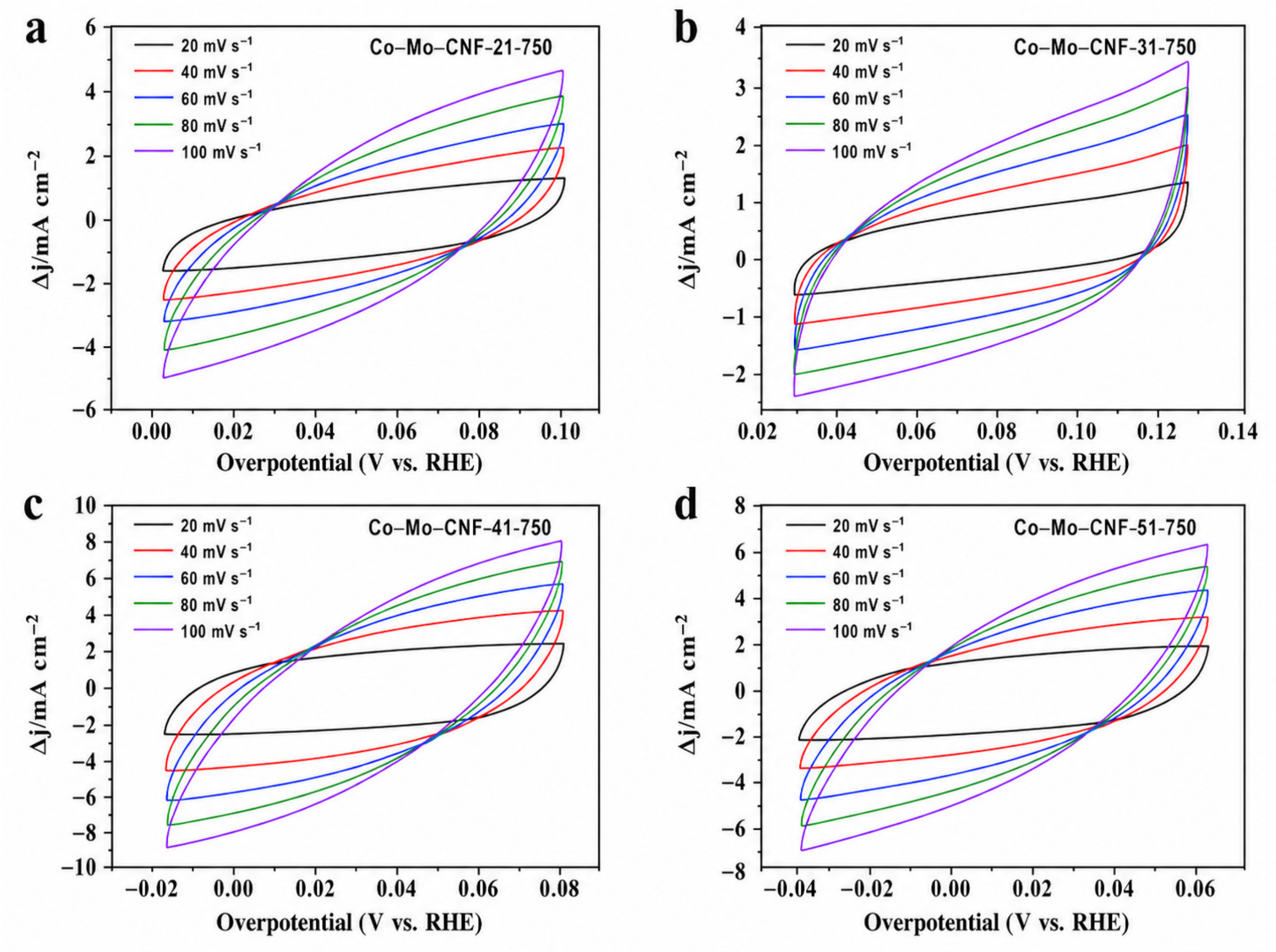

Figure S1. Cyclic voltammetry (CV) curves of P-free Co-Mo-CNF samples with different Co/Mo feed molar ratios. (a–d) Co-Mo-CNF-21-750, Co-Mo-CNF-31-750, Co-Mo-CNF-41-750, and Co-Mo-CNF-51-750, respectively, recorded in the non-Faradaic region at scan rates of 20, 40, 60, 80, and 100 mV s^{-1}.

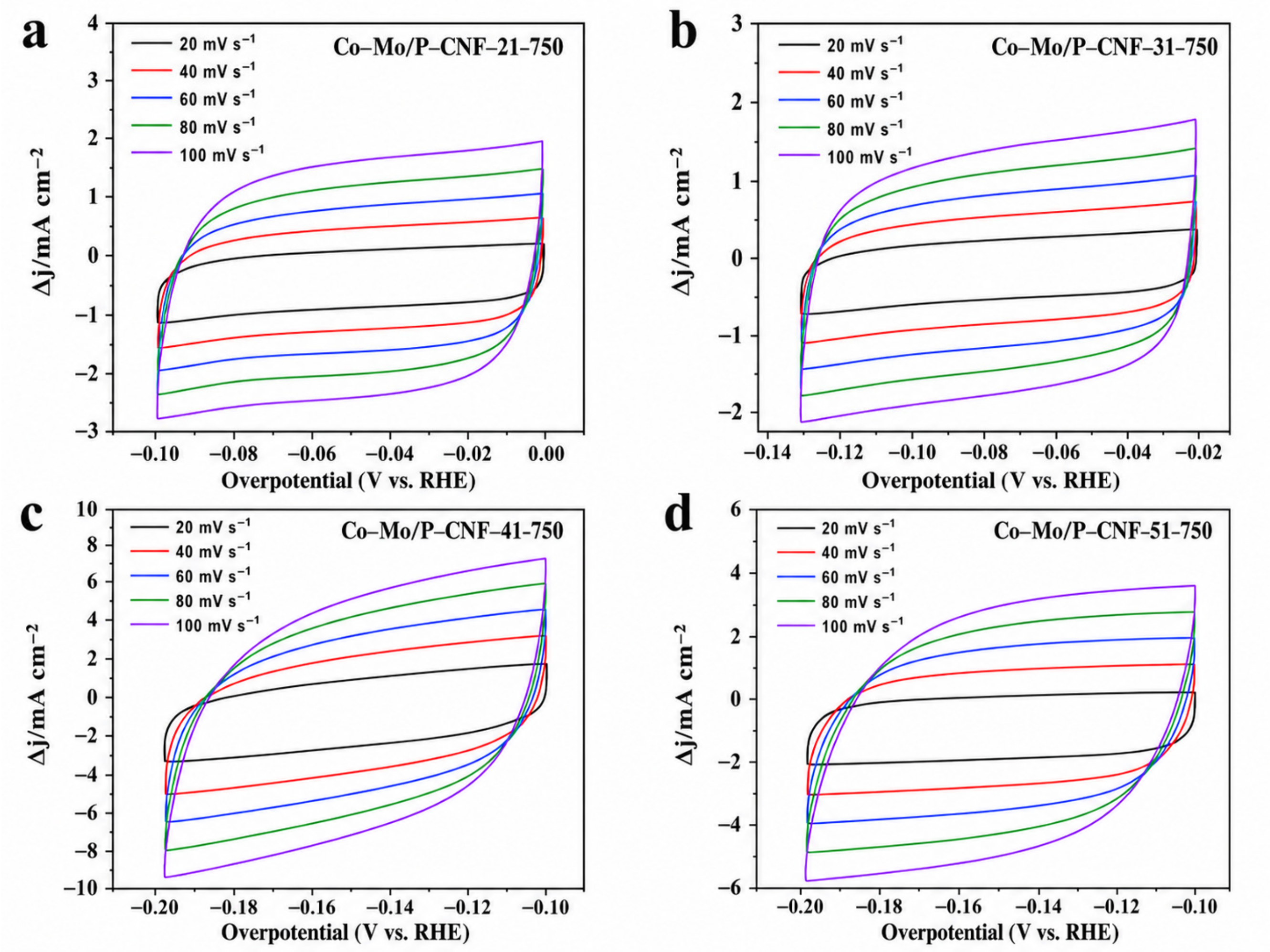

Figure S2. Cyclic voltammetry (CV) curves of P-containing Co-Mo/P-CNF samples with different Co/Mo feed molar ratios. (a–d) Co-Mo/P-CNF-21-750, Co-Mo/P-CNF-31-750, Co-Mo/P-CNF-41-750, and Co-Mo/P-CNF-51-750, respectively, recorded in the non-Faradaic region at scan rates of 20, 40, 60, 80, and 100 mV s^{-1}.

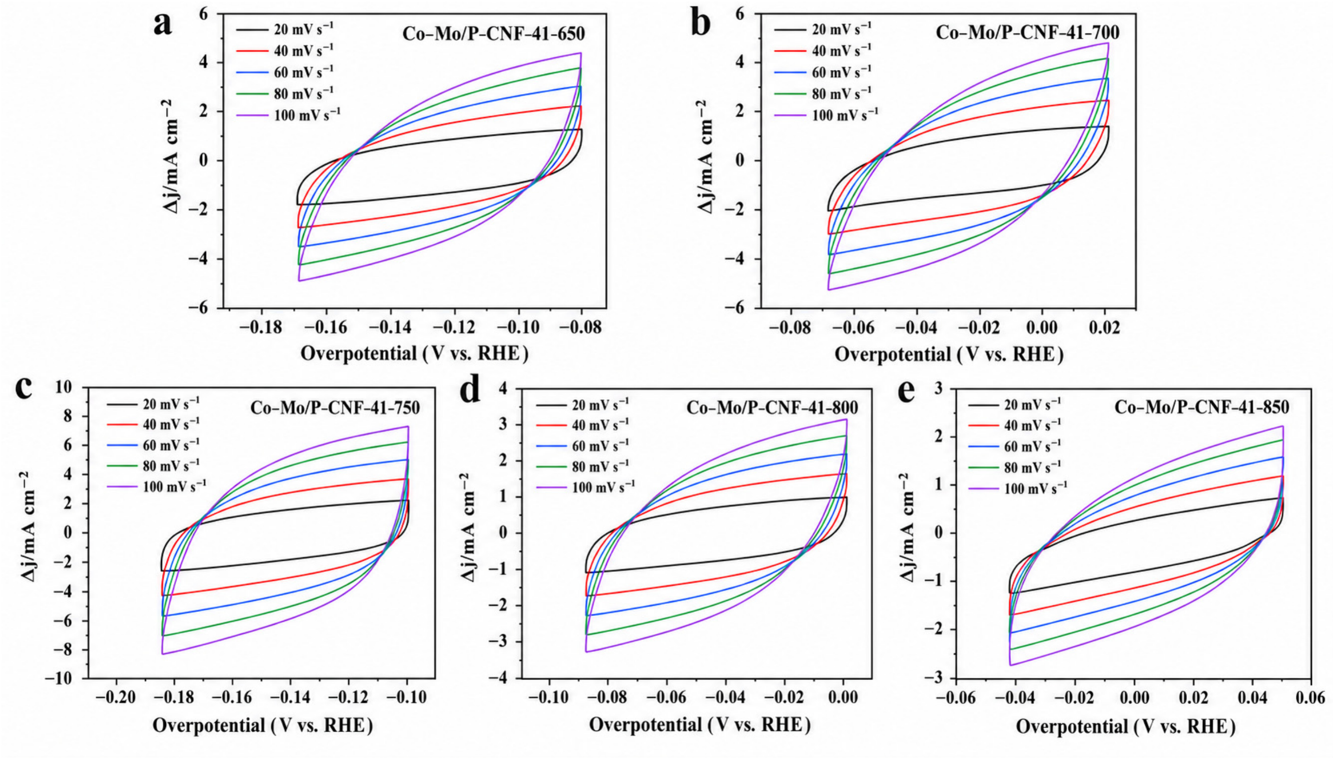

Figure S3. Cyclic voltammetry (CV) curves of pre-oxidized Co-Mo/P-CNF-41 samples carbonized at different temperatures. (a–e) Co-Mo/P-CNF-41-650, Co-Mo/P-CNF-41-700, Co-Mo/P-CNF-41-750, Co-Mo/P-CNF-41-800, and Co-Mo/P-CNF-41-850, respectively, recorded in the non-Faradaic region at scan rates of 20, 40, 60, 80, and 100 mV s^{-1}.

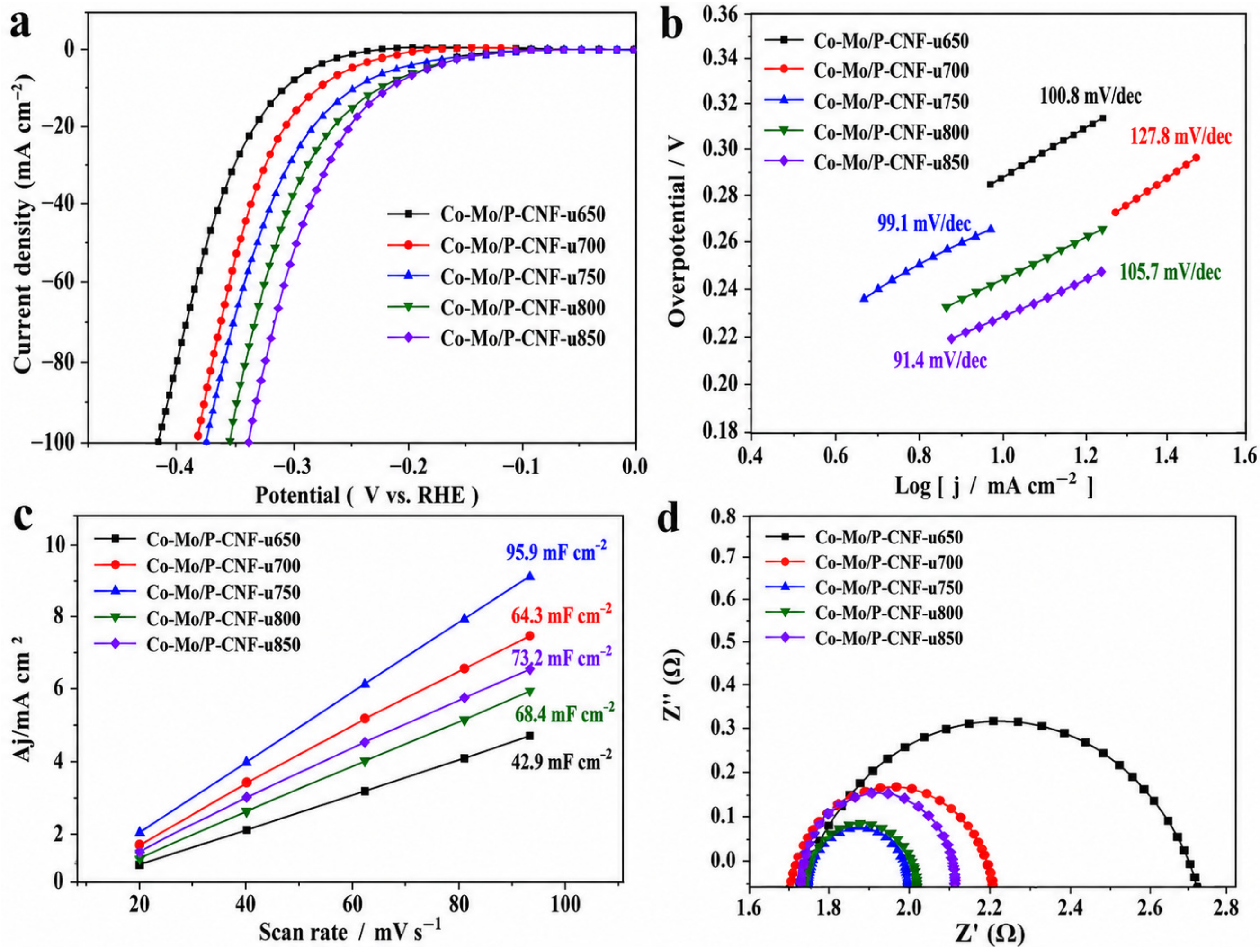

Figure S4. Full electrochemical comparison of Co-Mo/P-CNF-41 samples directly carbonized without pre-oxidation at 650–850 °C. (a) iR-corrected LSV curves; (b) Tafel plots; (c) C_{dl} fitting plots; and (d) Nyquist plots. For compact presentation, the plot labels Co-Mo/P-CNF-u650, Co-Mo/P-CNF-u700, Co-Mo/P-CNF-u750, Co-Mo/P-CNF-u800, and Co-Mo/P-CNF-u850 correspond to Co-Mo/P-CNF-41-u650, Co-Mo/P-CNF-41-u700, Co-Mo/P-CNF-41-u750, Co-Mo/P-CNF-41-u800, and Co-Mo/P-CNF-41-u850, respectively, as defined in the main manuscript.

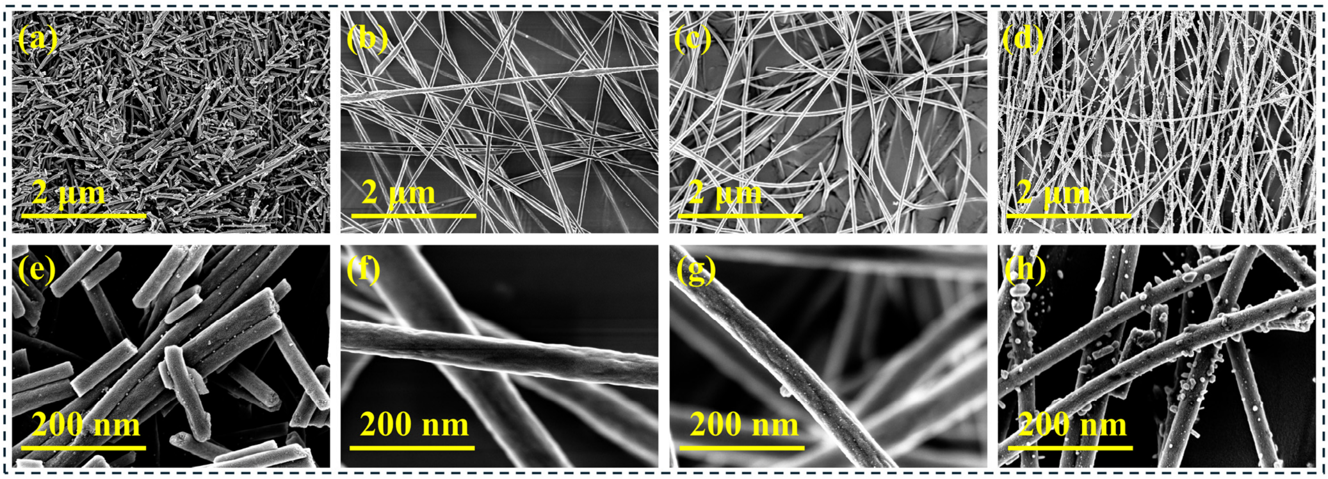

Figure S5. Low- and high-magnification SEM images of Co-Mo/P-CNF-41 samples directly carbonized without pre-oxidation at different temperatures. Panels (a–d) and (e–h) show the low- and corresponding high-magnification images, respectively: (a, e) Co-Mo/P-CNF-41-u650; (b, f) Co-Mo/P-CNF-41-u700; (c, g) Co-Mo/P-CNF-41-u800; and (d, h) Co-Mo/P-CNF-41-u850. The corresponding SEM image of Co-Mo/P-CNF-41-u750 is presented in Figure 4f of the main manuscript.

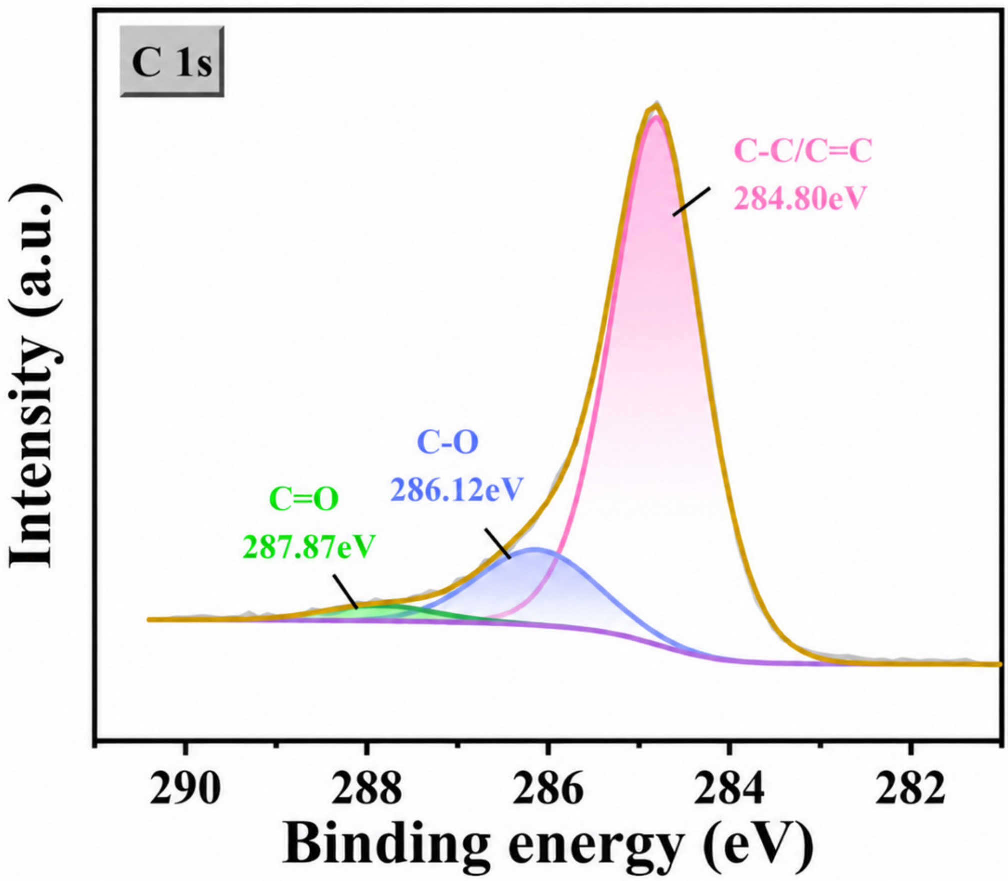

Figure S6. High-resolution XPS C 1s spectrum of Co-Mo/P-CNF-41-750. The C 1s spectrum was fitted with C–C/C=C (284.80 eV), C–O (286.12 eV), and C=O (287.87 eV) components.
